# Sex Differences in Clinical Features, Treatment, and Lifestyle Factors in Patients With Cluster Headache

**DOI:** 10.1212/WNL.0000000000201688

**Published:** 2023-03-21

**Authors:** Carmen Fourier, Caroline Ran, Anna Steinberg, Christina Sjöstrand, Elisabet Waldenlind, Andrea Carmine Belin

**Affiliations:** From the Departments of Neuroscience (C.F., C.R., A.C.B.), and Clinical Neuroscience (A.S., C.S., E.W.), Karolinska Institutet; Department of Neurology (A.S., E.W.), Karolinska University Hospital; and Department of Neurology (C.S.), Danderyd Hospital, Stockholm, Sweden.

## Abstract

**Background and Objectives:**

Cluster headache is considered a male-dominated disorder, but we have previously suggested that female patients may display a more severe phenotype. Studies on sex differences in cluster headache have been conflicting; therefore, this study, with the largest validated cluster headache material at present, gives more insights into sex-specific characteristics of the disease. The objective of this study was to describe sex differences in patient demographics, clinical phenotype, chronobiology, triggers, treatment, and lifestyle in a Swedish cluster headache population.

**Methods:**

Study participants were identified by screening medical records from 2014 to 2020, requested from hospitals and neurology clinics in Sweden for the *ICD-10* code G44.0 for cluster headache. Each study participant answered a detailed questionnaire on clinical information and lifestyle, and all variables were compared with regard to sex.

**Results:**

A total of 874 study participants with a verified cluster headache diagnosis were included. Of the participants, 575 (66%) were male and 299 (34%) were female, and biological sex matched self-reported sex for all. Female participants were to a greater extent diagnosed with the chronic cluster headache subtype compared with male participants (18% vs 9%, *p =* 0.0002). In line with this observation, female participants report longer bouts than male participants (*p* = 0.003) and used prophylactic treatment more often (60% vs 48%, *p =* 0.0005). Regarding associated symptoms, female participants experienced ptosis (61% vs 47%, *p =* 0.0002) and restlessness (54% vs 46%, *p =* 0.02) more frequently compared with male participants. More female than male study participants had a positive family history of cluster headache (15% vs 7%, *p =* 0.0002). In addition, female participants reported diurnal rhythmicity of their attacks more often than male participants (74% vs 63%, *p =* 0.002). Alcohol as a trigger occurred more frequently in male participants (54% vs 48%, *p =* 0.01), whereas lack of sleep triggering an attack was more common in female participants (31% vs 20%, *p =* 0.001).

**Discussion:**

With this in-depth analysis of a well-characterized cluster headache population, we could demonstrate that there are significant differences between male and female participants with cluster headache, which should be regarded at the time of diagnosis and when choosing treatment options. The data suggest that female patients generally may be more gravely affected by cluster headache than male patients.

Cluster headache is a severely painful primary headache disorder characterized by unilateral, orbitally located head pain and is often accompanied by autonomic symptoms, such as lacrimation or ptosis, or a feeling of restlessness. During an active bout, headache attacks occur up to 8 times per day and last between 15 and 180 minutes. In patients with episodic cluster headache, active bouts are separated by symptom-free remission periods, whereas in patients with chronic cluster headache, these remissions last less than 3 months per year.^1^ As many as 1 per 500 is estimated to have cluster headache, and although it is considered a male-dominated disease, the reported male-to-female ratio has shifted over the years from about 6:1 before 1960 to 5.1–2.5:1 in 2018.^2-5^ This shift is suggested to be an effect of lifestyle changes in both male and female patients, but possibly also due to increased recognition of the disease.^6^

A prominent feature of cluster headache is the striking diurnal and seasonal rhythmicity. For many patients, a cluster bout can arise during a certain time of the year while headache attacks recur with clock-like regularity at the same time of the day, leading to the premise that cluster headache is partly a disease of hypothalamic function and the circadian system.^7^ The hypothalamus displays a large variety of functions, such as regulation of metabolic and neuroendocrine processes and sleep and circadian rhythm.^8^ The suprachiasmatic nucleus (SCN), located within the hypothalamus, is considered the master clock of the brain orchestrating the daily cycles of behavior and physiology by synchronizing the peripheral circadian clocks of the body.^9^ The peripheral clocks are regulated on a molecular level by the so-called clock genes, including circadian locomotor output cycles kaput (*CLOCK*), which has been associated with cluster headache.^10^ In addition, studies could detect alterations in hormone levels for patients with cluster headache, including melatonin, supporting a hypothalamic involvement in cluster headache.^11,12^ These findings also pose the question how male and female patients with cluster headache are affected differently by the disease, specifically due to structural and functional sex differences in the hypothalamus.^13,14^ Of interest, the diurnal attack cycle is advanced by 1 hour in male compared with female patients with cluster headache, suggesting sex differences in cluster headache chronobiology.^15^

The literature is conflicting when comparing the clinical presentation of cluster headache in male and female patients. Although some studies could not see major differences in clinical characteristics between the sexes, others have observed discrepancies in age at onset, pain location, attack duration, associated symptoms, and comorbid conditions, such as depression.^4,15-18^

Because there appears to be a clear divergence for various aspects of cluster headache in male and female participants, we performed an in-depth analysis of a large validated cluster headache cohort. We examined and compared not only clinical phenotype and patterns of chronobiology but also heredity, treatment, trigger, and lifestyle factors between male and female patients with cluster headache from Sweden.

## Methods

### Study Population

All included study participants were diagnosed with cluster headache and subtype by neurologists through structured interviews and clinical examination, according to the criteria of *ICHD-3*.^1^ Study participants were identified between 2014 and 2020 by requesting medical records for the *ICD-10* code G44.0 for cluster headache from all major hospitals and neurology clinics over Sweden, as well as in conjunction with cluster headache patient visits to outpatient clinics. Four hundred thirty of the participants were diagnosed by the experienced neurologists and coauthors A. Steinberg, C. Sjöstrand, or E. Waldenlind at the Karolinska University Hospital. All medical records from G44.0 patients outside the Karolinska University Hospital catchment area (n = 444) were read by coauthors A. Steinberg, C. Sjöstrand, or E. Waldenlind to verify that the diagnosis fulfilled the criteria of the *ICHD-3*.^1^ Patients identified outside the Karolinska University Hospital catchment area were recruited by being contacted by phone and/or mail and asked to complete a paper questionnaire. For patients attending outpatient clinics, the questionnaire was completed in relation to the visit. This study is focused on differences between biological sex (male/female) with regard to cluster headache. All participants have been genotyped for their biological sex, and it matched their self-reported sex.

### Questionnaire

The questionnaire was composed of 3 parts consisting of both checklist and free text items (Table 1). The first part included personal, demographic, and medical information, the second part included questions designed to assess different clinical aspects of the disease, and the third part included questions related to lifestyle. Question 18a-c on annual rhythmicity and question 22 on pain intensity were added 2016 and were therefore not answered by all participants. Nonresponders were reminded up to 2 times to participate in the study. All questionnaire data are self-reported except cluster headache diagnosis, which was validated according to the criteria of the *ICHD-3*. All assessed variables compared with regard to sex are listed in Table 2.

**Table 1:**
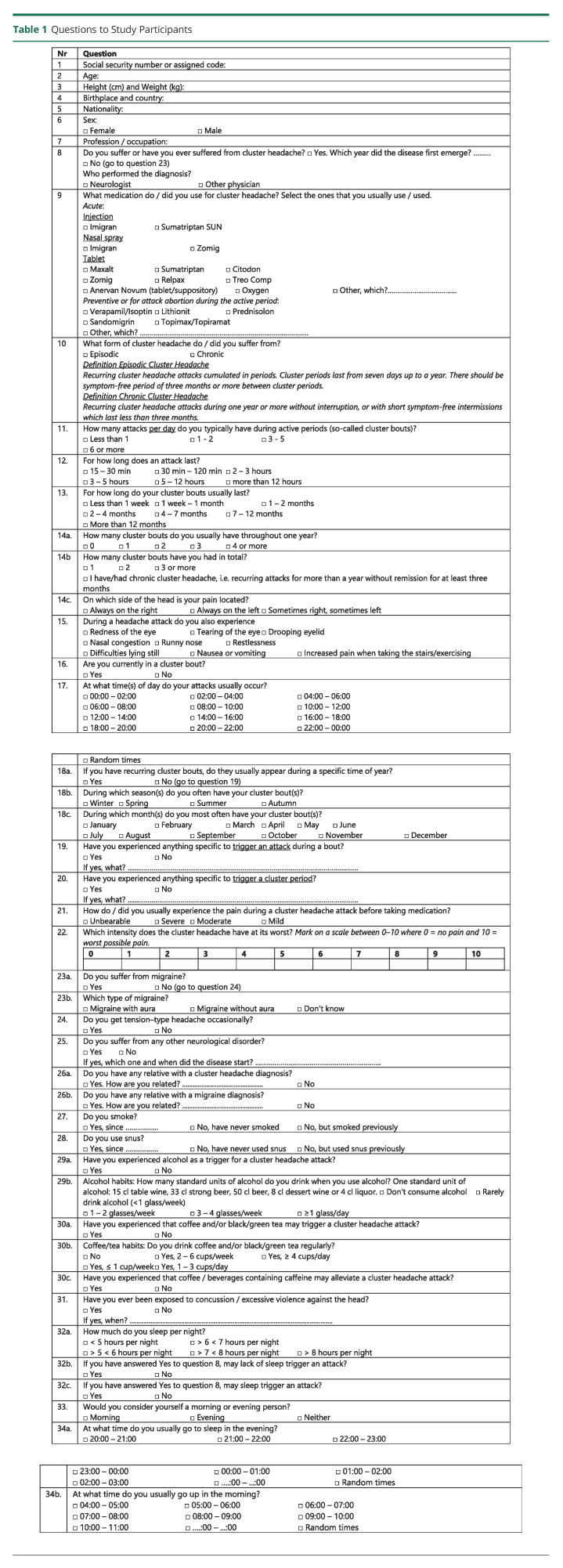
Questions to Study Participants

**Table 2:**
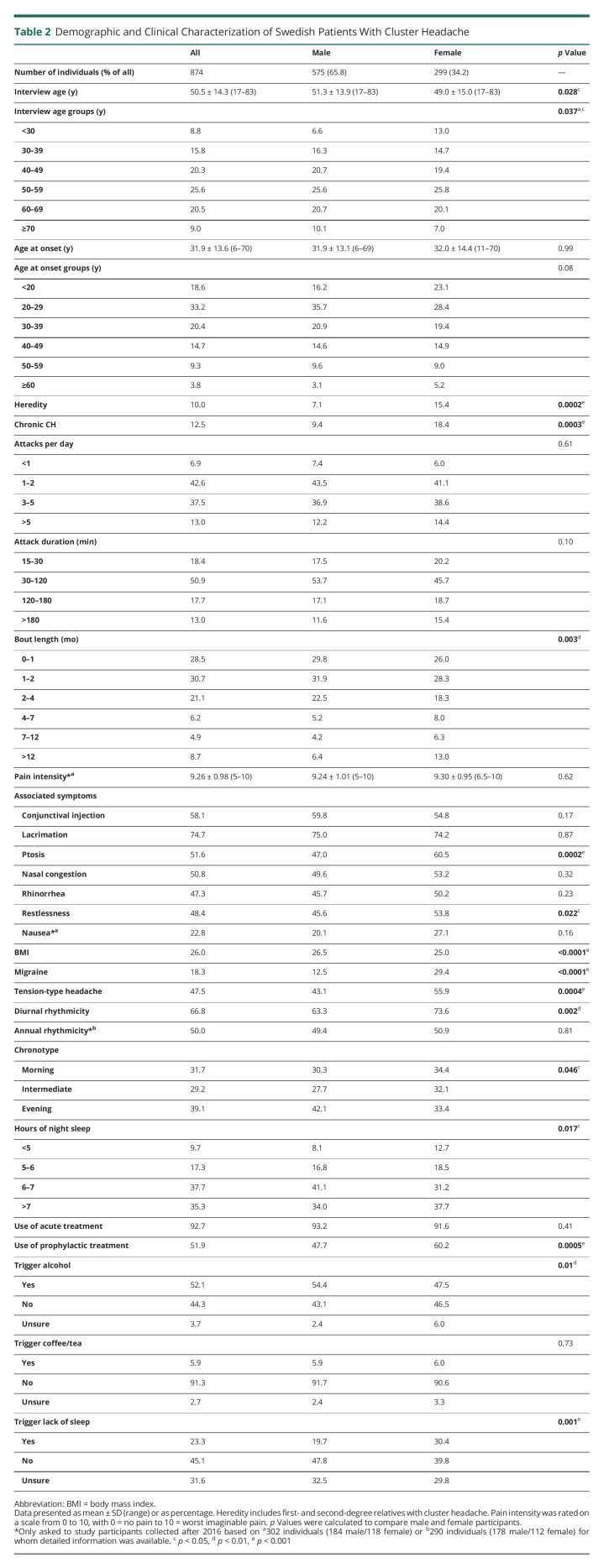
Demographic and Clinical Characterization of Swedish Patients With Cluster Headache

### Statistical Analysis

The Welch *t* test was used to compare the means of continuous variables. The Fisher exact test or Chi-square (χ^2^) test for more than 2 categories was used to compare the proportions of categorical variables. All tests were 2 tailed, and α < 0.05 was considered statistically significant. Analyses were performed using GraphPad Prism version 9.0.0 for Windows (GraphPad Software, San Diego, CA, graphpad.com). All data are presented as either mean ± SD or as proportions with odds ratio (OR) and 95% CI for male vs female participants.

### Standard Protocol Approvals, Registrations, and Patient Consents

The study was approved by the Swedish Ethical Review Authority in Stockholm, Sweden (diary number 2014/656-31/4). Written informed consent was obtained from all participants in the study.

### Data Availability

Anonymized data not published within this article will be made available by request from any qualified investigator following the Karolinska Institutet data transfer agreement and General Data Protection Regulation.

## Results

**Figure 1:**
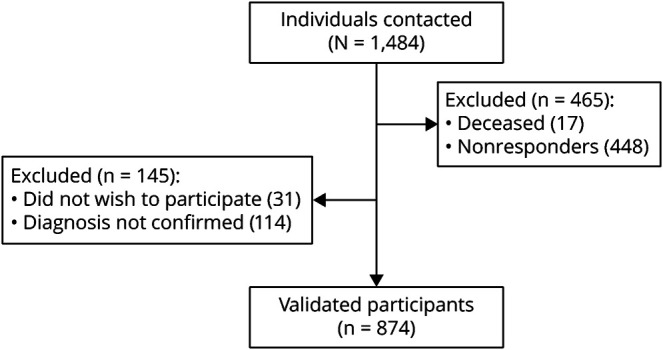
Flow Diagram Showing Inclusion and Exclusion of Patients With Cluster Headache for Study Participation A total of 1,484 individuals were recruited for inclusion in our cluster headache biobank. Four hundred ninety-six were excluded due to the following: deceased before study start (n = 17), did not wish to participate (n = 31), or had not replied at the specific time point of data collection (n = 448). A G44.0 diagnosis could not be confirmed in 114, and in total, 874 study participants validated with a G44.0 diagnosis participated in this questionnaire study.

To date, 1,484 individuals were recruited for inclusion in our cluster headache biobank, of which 874 participated in this questionnaire study (Figure 1). Of note, 496 individuals were excluded due to the following: deceased before study start (n = 17), did not wish to participate (n = 31), or had not replied at the specific time point of data collection (n = 448), of which 293 (65.4%) were male and 155 (34.6%) were female. Of the remaining 988 individuals, a G44.0 diagnosis could not be confirmed for 114. Of the 874 participants, all validated with a G44.0 diagnosis by the authors (A.S., C.S., or E.W.) according to the *ICHD-3* criteria, 575 (65.8%) were male and 299 (34.2%) were female. One hundred eighty-six (21.9%) reported to be in a cluster bout when answering the questionnaire. No individuals were removed due to missing data (eTable 1, links.lww.com/WNL/C534). Demographic and clinical data and the subsequent statistics have been summarized in Table 2. The age at the time of completing the questionnaire differs slightly between male and female participants (51.3 ± 13.9 vs 49.0 ± 15.0, *p* = 0.028). Male and female participants do not differ in age at cluster headache onset, although there is a lower proportion of male participants with cluster headache onset below the age of 20 years compared with female participants (16.2% vs 23.0%, OR [95% CI]:0.64 [0.44–0.92], *p* = 0.020). We have previously reported that patients with chronic cluster headache have a later mean disease onset than patients with episodic cluster headache, and this delay could be observed in both sexes in the present study (eFigure 1A, links.lww.com/WNL/C534).^19^ Regarding family history, significantly fewer male participants than female participants had a first- or second-degree relative also diagnosed with cluster headache (7.1% vs 15.4%, OR [95% CI]:0.42 [0.27–0.66], *p =* 0.0002).

Of interest, the proportion of male participants with chronic cluster headache was lower than for female participants (9.4% vs 18.4%, OR [95% CI]:0.46 [0.31–0.70], *p* = 0.0002). In line with this observation, bout length, but not attack frequency or attack duration, differed between male and female participants (*p* = 0.004), with female participants tending to have longer cluster headache bouts than male participants. Pain intensity was reported equally high for both sexes, and associated symptoms occurred similarly except for ptosis (47.0% vs 60.5%, OR [95% CI]:0.58 [0.43–0.76], *p =* 0.0002) and restlessness (45.6% vs 53.9%, OR [95% CI]:0.72 [0.54–0.95], *p* = 0.024), which were both more common in participants. The mean body mass index (BMI) differed significantly between male and female participants (26.5 ± 4.1 vs 25.0 ± 4.9, *p <* 0.0001). In addition, fewer male than female participants have self-reported migraine (12.5% vs 29.4%, OR [95% CI]:0.34 [0.24–0.49], *p <* 0.0001), and male participants suffered less from tension-type headache than female participants (44.3% vs 57.6%, OR [95% CI]:0.58 [0.44–0.78], *p =* 0.0002).

**Figure 2:**
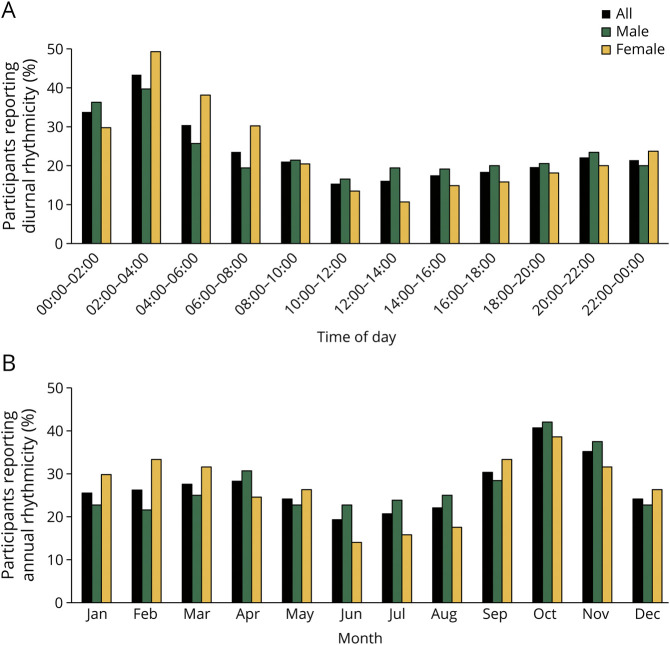
Diurnal and Annual Reoccurrence of Cluster Headache Attacks and Bouts (A) Participant frequency of attack distributions over 24 hours in 2-hour intervals for patients reporting diurnal rhythmicity. Data from 565 participants with cluster headache (350 male/215 female). Attack distribution by time of day differs significantly between male and female participants (*p =* 0.002). (B) Participant frequency of bout distribution over the year in months for patients reporting annual rhythmicity. Data from 145 participants with cluster headache (88 male/57 female). Bout distribution by month did not differ between male and female participants (*p =* 0.72).

The presence of diurnal rhythmicity of attacks was less common in male than female participants with cluster headache (364/575 [63.3%] vs 220/299 [73.6%], OR [95% CI]:0.62 [0.46–0.85], *p =* 0.002). In addition, the number of attacks per 2-hour time interval over 24 hours differed between male and female participants (*p =* 0.002), with a higher frequency of nightly attacks in female participants (Figure 2A). We could not find a difference in the occurrence of annual rhythmicity of cluster headache bouts or in the bout distribution by month of the year between the sexes (Figure 2B). Male and female participants differed slightly in self-reported chronotype, morning, evening, or neither. Female participants tended to be morning types, whereas more male participants tended to describe themselves as evening types (*p =* 0.046). Because a shift in chronotype with age has been reported, we also analyzed chronotype in different age groups between male and female participants (eTable 2, links.lww.com/WNL/C534).^20,21^ Overall, we observed a difference in chronotypes by sex and age (*p =* 0.001). In addition, hours of night sleep varied between male and female participants (*p =* 0.017), especially more female participants had less than 5 hours of sleep per night.

**Figure 3:**
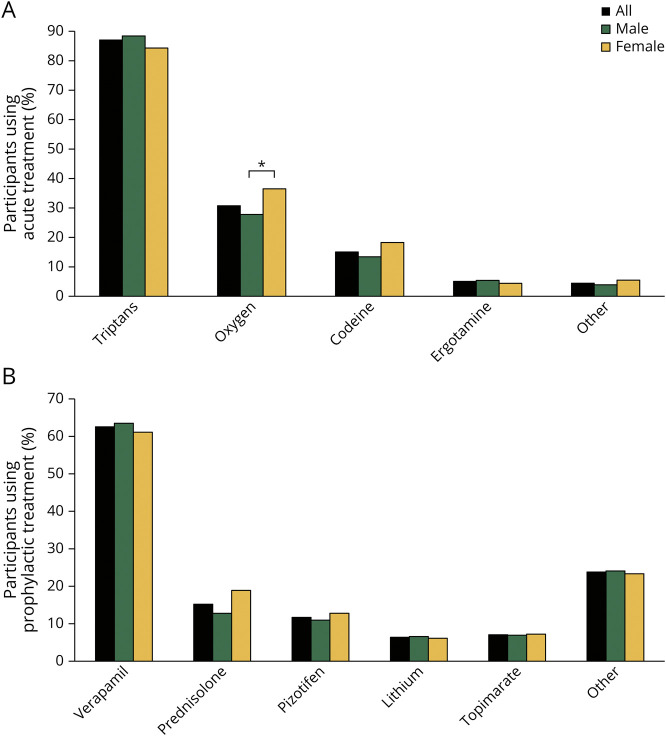
Acute and Prophylactic Treatments in Participants With Cluster Headache (A) Most used medication for attack abortion among participants who report using acute treatment. Data from 810 participants with cluster headache (536 male/274 female). *Oxygen was more commonly used by female participants compared with male participants (*p* = 0.013). (B) Most used preventive medication among participants with cluster headache who report using prophylactic treatment (n = 454, 274 male/180 female).

When comparing acute and prophylactic treatments between male and female participants, both sexes generally used abortive medication at equally high proportions (>90% of the participants). In addition, we specifically compared different abortive medications (Figure 3A) and detected a less frequent use of oxygen by male participants compared with female participants (27.8% vs 36.5%, OR [95% CI]:0.67 [0.49–0.92], *p =* 0.013). Oxygen is not prescribed to smokers in Sweden, and it has been reported that patients with cluster headache (both male and female participants) smoke more than the general population.^19^ However, there was no difference in smoking habits between male and female participants (26.8% vs 25.1%, *p =* 0.63; eTable 3, links.lww.com/WNL/C534). Regarding prophylactic treatment, significantly fewer male participants used preventive medication than did female participantsma (47.7% vs 60.2%, OR [95% CI]:0.60 [0.45–0.80], *p =* 0.0005), although the use of specific prophylactic medications did not differ between the sexes (Figure 3B).

**Figure 4:**
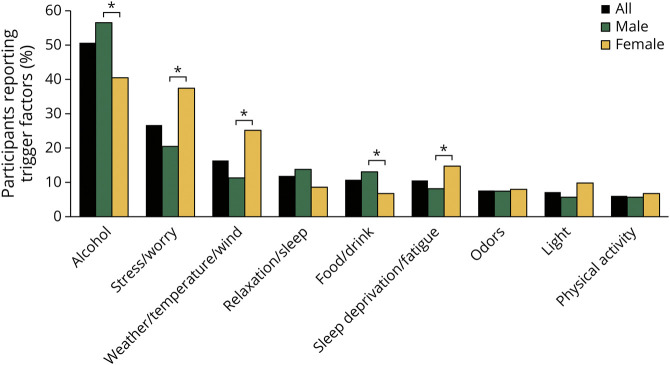
Most Common Trigger Factors for Cluster Headache Attacks During a Bout for Study Participants Reporting Specific Triggers (Free-Text Answers) Four hundred forty-six participants with cluster headache (283 male/163 female) reported yes to specific trigger factors. *Significant differences between male and female participants who report trigger factors were found for alcohol (*p* = 0.001), stress/worry (*p* = 0.0001), weather/temperature/wind (*p* = 0.0003), food/drink (*p* = 0.040), and sleep deprivation/fatigue (*p* = 0.037).

In our questionnaire, we asked participants to list any possible triggers that could elicit a cluster headache attack during a bout in a free-text answer, and we categorized and summarized the most common answers for all participants, subdivided by sex, in Figure 4. There was no significant difference in reporting trigger factors as free-text answers between male and female participants (49.2% vs 54.5%, *p =* 0.15, data not shown). Alcohol was by far the most listed trigger among participants who reported trigger factors for their attacks (50.7%), and male participants reported more often than female participants that alcohol provoked a cluster headache attack (56.5% vs 40.5%, OR [95% CI]:1.91 [1.29–2.81], *p =* 0.001). Alcohol intake differed significantly between male and female participants, with male participants generally consuming more alcohol than female participants (*p* < 0.0001; see eTable 4, links.lww.com/WNL/C534). This trend between sexes is also seen in the general Swedish population (source: Statistics Sweden), although it appears that overall fewer participants have a high alcohol consumption, whereas more participants have seldom or no alcohol consumption compared with the general population. Of interest, we could not see a difference in alcohol intake (*p =* 0.25) or alcohol as trigger factor (*p =* 0.67, data not shown) when comparing male and female participants with chronic cluster headache. The second most described trigger factor for cluster headache attacks was stress (26.7%), which was not as common for male participants as for female participants (20.5% vs 37.4%, OR [95% CI]:0.43 [0.28–0.66], *p =* 0.0001). Other trigger factors that differed significantly between male and female participants were changes in weather/temperature or draft/gust (11.3% vs 25.2%, OR [95% CI]:0.38 [0.23–0.63], *p =* 0.0003), lack of sleep (8.1% vs 14.7%, OR [95% CI]:0.51 [0.28–0.92], *p =* 0.037), and food/drink (13.1% vs 6.7%, OR [95% CI]:2.08 [1.06–4.20], *p =* 0.040). Most described food items or nonalcoholic beverages that may trigger an attack were chocolate, sweets or food/beverages with high sugar content, coffee/tea, (strong) cheese, food with high salt content, and spicy/pungent food. In addition to free-text answers, participants with cluster headache were specifically asked whether alcohol, coffee/tea, or lack of sleep may trigger an attack. These specific questions confirmed the free-text answers: Alcohol as a trigger was more commonly reported by male participants (*p =* 0.01), whereas lack of sleep triggered attacks in female participants more often than in male participants (*p =* 0.001). There was no difference for coffee/tea as a trigger, and 1.2% of the female participants reported hormone related factors, like menstruation, as a trigger.

## Discussion

In this study, we methodically analyzed and compared demographic, clinical, and lifestyle features between 575 male and 299 female participants with cluster headache from Sweden. We did not find a difference in mean age at onset between male (31.9 years) and female (31.8 years) participants, but a higher proportion of female participants with cluster headache onset below the age of 20 years, which agrees with a study on cluster headache in the United States.^18^ It is unclear why more female than male participants had an early onset of cluster headache. Of interest, both male and female participants with chronic cluster headache showed a delay in disease onset with no difference between the groups (data not shown), except for a minor second peak at onset above 60 years. These data need to be interpreted with care due to thinning down of the sample size but could suggest that female patients with late cluster headache onset have a harsher form of the disease. Overall, we could not confirm a bimodal pattern for disease onset as reported in the American study, only an indication of 2 peaks for female participants with chronic cluster headache, which has also been suggested in an Italian study.^22^ This bimodal pattern has led to speculations on hormonal involvement in cluster headache, as these peaks coincide with menarche and menopause in female participants.^16^ However, a study on the influence of, for example, menstruation and menopause could not find a clear association with cluster headache.^23^

It was previously observed in a Danish study that both male and female patients with cluster headache have a higher mean BMI compared with controls.^24^ In our study, BMI categories of male and female participants with cluster headache were compared with the general Swedish population using publicly available health data from Statistics Sweden (eTable 5, links.lww.com/WNL/C534). The mean BMI was higher for male participants with cluster headache than for female participants (*p* < 0.0001), which is also seen in the general population (*p* < 0.0001). When comparing with the general population, we found a higher mean BMI for cluster headache (*p <* 0.0001). Of interest, this difference was only significant for male participants (*p* = 0.009) but not for female participants (*p* = 0.34). When analyzing the frequencies of the different BMI categories (underweight, normal, overweight, and obese) in participants and controls, we observed the same trend: male, but not female, participants tend to be more overweight or obese compared with the general male (*p* = 0.005) and female (*p* = 0.66) populations. This suggests that especially male patients with cluster headache have a risk of being overweight, possibly due to a less healthy lifestyle. Curiously, significantly more female (15%) than male (7%) participants had a first- or second-degree relative also diagnosed with cluster headache, which is in concordance with 2 recently published review articles on family history of cluster headache where they noted a predominance of female patients with familial cluster headache.^25,26^

In addition, there was a preponderance of female participants (18%) over male participants (10%) with the chronic cluster headache subtype, which has been observed in another Scandinavian cohort recently.^27^ This is also reflected in longer cluster headache bouts for female participants compared with male participants, which indicates that although cluster headache is still considered a predominantly male disease, female participants may generally have a more debilitating form of cluster headache. Nevertheless, the pain intensity during a cluster headache attack does not differ between sexes and is equally excruciating for both male and female patients, as previously reported.^4,15^

Generally, we could see that associated symptoms occurred with similar frequencies in male and female participants. Only ptosis and restlessness were more common in female participants, which agrees with earlier studies (note that restlessness was phrased as need to move).^15,16^ We could not confirm nausea to be more frequent in female participants with cluster headache, nor was nausea associated with migraine in our cluster headache cohort (data not shown).^16,18,28^

The prevalence of migraine is found to be between 13.8% and 18% in Sweden, with a majority of the affected being female.^29,30^ As in the general population, fewer male than female participants with cluster headache have self-reported migraine (*p <* 0.0001). Depending on which study we compare our data with, we could or could not find differences in the prevalence of migraine between the general Swedish population and Swedish participants with cluster headache (eTable 6, links.lww.com/WNL/C534). When comparing with Swedish twins in the PILOT study, both male and female participants with cluster headache have a higher prevalence for migraine than controls.^29^ This difference could not be seen in comparison to the OCTO-Twin or the GENDER study of Swedish twins except for female participants in our cluster headache cohort compared with female participants in the OCTO-Twin study.^30^ As for other primary headache disorders, male patients with cluster headache suffered less from tension-type headache than female patients (*p =* 0.0002). In the PILOT study, the prevalence of tension-type headache for the general Swedish population is 10.9% for male participants and 15.7% for female participants, which is quite a bit lower than in our cluster headache cohort (*p <* 0.0001, data not shown).^29^ However, population studies from other countries have found a higher prevalence of tension-type headache, which indicates a high variability.^31,32^ Because these data are not based on a clinical diagnosis but are self-reported, we need to be careful with interpreting these results. The Danish Headache Centre reported a high comorbidity of clinically diagnosed tension-type headache in patients with migraine (67%); thus, there may indeed be an increased incidence of other types of headaches in patients with primary headache.^33^ To our knowledge, little is known on comorbidity of tension-type headache with cluster headache. Our findings suggest that female patients with cluster headache are more likely to have other primary headache disorders in addition to cluster headache.

Chronobiology clearly differs between male and female patients with cluster headache. More female (74%) than male (63%) participants report diurnal rhythmicity of their attacks, and although both sexes have a peak of attacks occurring at nighttime, female participants were more likely to have cluster headache attacks at night and during the early morning hours, whereas the lowest frequency of attacks for female participants was around lunchtime. For male participants, attack occurrence was more spread out throughout the 24-hour day. Annual rhythmicity of cluster bouts occurred roughly in 50% of both sexes, which agrees with previous observations.^15^ The distribution of cluster bouts throughout the year shows a clear peak in fall and a smaller peak in spring for both sexes. A study on an arctic cluster headache population found that cluster bouts more likely started around the equinoxes in March and September, times of the year when the daily change of natural light is most noticeable and the SCN increases in size.^34,35^ Intriguingly, a preliminary study from Portugal could find an increase in CLOCK gene expression in patients with cluster headache at the September equinox.^36^ These findings suggest an involvement and possible dysregulation of circadian rhythm in cluster headache producing specific diurnal and annual attack patterns.

Nocturnal sleep duration differed significantly between male and female participants. Most male participants slept 6–7 hours per night, whereas female participants had a large variance in sleep duration. A higher proportion of female participants (13%) slept less than 5 hours per night compared with male participants (8%), which may be explained by the more frequent nighttime cluster headache attacks. Studies on sex differences for sleep in the general population report longer sleep duration for female participants.^37^ In addition, more female than male participants report inadequate sleep, which, together with the observation that male persons more likely function well with less than 7 hours of sleep, suggests that female persons may be more susceptible to clinical symptoms from sleep difficulties and could potentially be more debilitated by nocturnal cluster headache attacks.^38,39^

With respect to treatment for cluster headache attacks, there was no difference in use of acute medication between male and female participants, except for oxygen, which was reported more frequently in female participants (37%). We could exclude smoking as a factor contributing to a lower use of oxygen in male participants (28%). It remains unclear why female participants with cluster headache in Sweden use oxygen more often than male participants to abort cluster headache attacks. Other studies rather report the opposite, and although efficacy does not seem to differ between the sexes, a slower response has been observed in female study participants.^40,41^ The use of prophylactic medication during cluster headache bouts was higher in female (60%) compared with male (48%) participants, which is not surprising, considering that significantly more female persons have chronic cluster headache.

To reduce bias, participants were asked to write down specific triggers for their cluster headache attacks during a bout instead of presenting them with a list of possible trigger factors. The response rate to this question was similar between male and female participants, therefore unlikely that one sex recalled possible triggers better than the other. Alcohol was the most common trigger to elicit an attack among those reporting triggers, and more male participants (57%) than female participants (40%) reported alcohol as a trigger, which concurs with previous reports.^15,18^ As to why this is, a possible explanation could be that male participants with episodic cluster headache consume more alcohol than female participants with episodic cluster headache and they may not reduce their alcohol intake as much during a bout as female participants. However, our data do not differentiate between alcohol consumption during and out of bout but rather reflects general intake. We could not directly compare alcohol intake between participants with cluster headache and the general Swedish population due to different definitions (eTable 6, links.lww.com/WNL/C534), but it appears that both male and female participants with cluster headache have a lower alcohol consumption than the general Swedish population, which would contradict a Danish study on lifestyle of patients with cluster headache.^24^ However, we think that a reduced alcohol intake in participants with cluster headache seems rational, considering that alcohol is a very common trigger for headache. In addition, our data suggest that participants with cluster headache with a high disease burden have very low alcohol consumption.

Other often reported trigger factors were stress and lack of sleep, which were both more frequently reported by female participants (38% and 15%) than by male participants (21% and 8%). Research has pointed out sex differences in stress response mechanisms. Female study participants have been reported to be more vulnerable to stress-induced hyperarousal, a state of increased agitation, restlessness, and sleep disruption, whereas male participants are more afflicted with stress-induced cognitive deficits and structural and functional changes in brain regions critical for cognition.^42^ Perhaps, stress-induced arousal may, among other things, lead to sleep deprivation and consequently contribute to triggering a cluster headache attack in female patients. This is also supported by the higher frequency of restlessness seen during attacks in female participants. In addition, when participants with cluster headache were specifically asked whether sleep deprivation elicits an attack, 31% of the female participants and only 20% of the male participants said yes, indicating that female patients with cluster headache are more sensitive to disrupted sleep. Of interest, a Korean study on sex differences reports higher perceived psychological stress in female compared with male participants with cluster headache.^4^

The causal relationship between attack triggers and cluster headache is still unclear, and some triggers may be associated wrongly. For example, both stress and relaxation (after stress) are mentioned as separate trigger factors, although they are interconnected. In addition, in migraine, many premonitory symptoms may be misinterpreted as triggers, for example, chocolate and the craving for sweets or chocolate.^43^ In cluster headache, although not as pronounced, premonitory symptoms have also been reported and could therefore underlie the same misinterpretation by participants.^44^

The findings of this study reveal distinct differences in chronobiology and disease burden between the sexes. Cluster headache in female participants presents with more pronounced diurnal rhythmicity and may affect female patients much more in their everyday life, as demonstrated by nightly attacks disrupting their sleep and overall longer cluster headache bouts leading to the need for more prophylactic treatment. In addition, female persons are more likely to have cluster headache if they have a family history of the disease, and female participants with cluster headache are more likely to have other headache disorders. Finally, female patients may be more susceptible to stress and sleep deprivation as triggers for their cluster headache attacks. Our study indicates that male patients generally had a less healthy lifestyle, underlined by a higher BMI and tobacco consumption than the general population and a higher alcohol intake than female patients.

The strengths of this study are the large sample size, and a well-defined and representative cohort where all participants have been diagnosed with cluster headache by a neurologist, according to the *ICHD-3*. Cluster headache prevalence was recently reported to be 0.054% in individuals of working age in Sweden.^45^ This corresponds to approximately 5,400 individuals, and more than 25% of these have been contacted to participate in this study. Participants were recruited via different channels (medical records and outpatient clinics), which may reduce the risk of only including participants with high disease burden. The sex distribution among nonresponders at the specific time point of data collection (65.4% male and 34.6% female) was similar to the responder group (65.8% male and 34.2% female), making it unlikely that results are influenced by differential participation by sex. There are limitations to our study. All data are self-reported, which may introduce recall bias in relation to, for example, medication and if the participant was in or out of bout. Our study did not include, and thereby did not consider, persons whose biological sex characteristics and self-reported sex are not synonymous. Sex bias in diagnosis could potentially contribute to the observed differences in severity. Cluster headache is still considered to be a male-dominated disorder and may thereby make it more difficult for female patients with milder symptoms to be diagnosed with cluster headache than male participants, which could contribute to the higher relative frequency of, for example, chronic cluster headache in the female population. In addition, this is an observational study where causality is difficult to infer from the associations found.

In conclusion, this is a large study on sex differences in verified cluster headache patients, which may help to increase our understanding in which manner the disorder manifests differently in male and female patients. Cluster headache is still often misdiagnosed in female patients, perhaps because certain features of the disease in female patients resemble a migraine-like phenotype. It is therefore of utmost importance for physicians to be aware of these sex differences when working in the clinic and meeting patients with headache to be able to give the most effective treatment as fast as possible.

## Glossary

CLOCK: Circadian Locomotor Output Cycles Kaput
GDPR: General Data Protection Regulation
*ICD-10*: *International Classification of Diseases, 10th revision*
*ICHD-3*: *International Classification of Headache Disorders, 3rd Edition*
SCN: suprachiasmatic nucleus

## References

[1] Headache Classification Committee of the International Headache Society (IHS). The International Classification of Headache Disorders, 3rd Edition. Cephalalgia. 2018;38(1):1-211. doi: 10.1177/0333102417738202. 29368949

[2] Ekbom K, Svensson DA, Pedersen NL, Waldenlind E. Lifetime prevalence and concordance risk of cluster headache in the Swedish twin population. Neurology. 2006;67(55):798-803. doi: 10.1212/01.wnl.0000233786.72356.3e 16966540

[3] Manzoni GC. Gender ratio of cluster headache over the years: a possible role of changes in lifestyle. Cephalalgia. 1998;18(33):138-142. doi: 10.1046/j.1468-2982.1998.1803138.x 9595206

[4] Chung P, Lee MJ, Park J, et al. Differences of cluster headache on the basis of sex in the Korean cluster headache registry. Headache. 2019;59(10):1722-1730. doi: 10.1111/head.13637 31535372

[5] Bahra A, May A, Goadsby PJ. Cluster headache: a prospective clinical study with diagnostic implications. Neurology. 2002;58(3):354-361. doi: 10.1212/WNL.58.3.354 11839832

[6] Goadsby P, Wei DT, Yuan Ong J. Cluster headache: epidemiology, pathophysiology, clinical features, and diagnosis. Ann Indian Acad Neurol. 2018;21(5):S3–S8. doi: 10.4103/AIAN.AIAN_349_17 29720812 PMC5909131

[7] Burish MJ, Chen Z, Yoo SH. Cluster headache is in part a disorder of the circadian system. JAMA Neurol. 2018;75(7):783-784. doi: 10.1001/jamaneurol.2018.1049 29800013 PMC6390835

[8] Lechan RM, Toni R. Functional Anatomy of the Hypothalamus and Pituitary. MDText.com, Inc.; 2000.

[9] Albrecht U. Timing to perfection: the biology of central and peripheral circadian clocks. Neuron. 2012;74(2):246-260. doi: 10.1016/j.neuron.2012.04.006 22542179

[10] Fourier C, Ran C, Zinnegger M, et al. A genetic CLOCK variant associated with cluster headache causing increased mRNA levels. Cephalalgia. 2018;38(3):496-502. doi: 10.1177/0333102417698709 28466652

[11] Stillman MJ. Testosterone replacement therapy for treatment refractory cluster headache. Headache. 2006;46(6):925-933. doi: 10.1111/j.1526-4610.2006.00436.x 16732838

[12] Leone M, Bussone G, Leone Bussone GM. A review of hormonal findings in cluster headache. Evidence for hypothalamic involvement. Cephalalgia. 1993;13(5):309-317. doi: 10.1046/J.1468-2982.1993.1305309.X 8242722

[13] Swaab DF, Chung WCJ, Kruijver FPM, Hofman MA, Hestiantoro A. Sex differences in the hypothalamus in the different stages of human life. Neurobiol Aging. 2003;24(Suppl 1):1-16. doi: 10.1016/S0197-4580(03)00059-9 12829102

[14] Delaruelle Z, Ivanova TA, Khan S, et al. Male and female sex hormones in primary headaches. J Headache Pain. 2018;19(1):117. doi: 10.1186/s10194-018-0922-7 30497379 PMC6755575

[15] Lund N, Barloese M, Petersen A, Haddock B, Jensen R. Chronobiology differs between men and women with cluster headache, clinical phenotype does not. Neurology. 2017;88(11):1069-1076. doi: 10.1212/WNL.0000000000003715 28202701

[16] Allena M, de Icco R, Sances G, et al. Gender differences in the clinical presentation of cluster headache: a role for sexual hormones? Front Neurol. 2019;10:1220. doi: 10.3389/fneur.2019.01220 31824403 PMC6882735

[17] Liang JF, Chen YT, Fuh JL, et al. Cluster headache is associated with an increased risk of depression: a nationwide population-based cohort study. Cephalalgia. 2013;33(3):182-189. doi: 10.1177/0333102412469738 23212294

[18] Rozen TD, Fishman RS. Female cluster headache in the United States of America: what are the gender differences? J Neurol Sci. 2012;317(1‐2):17-28. doi: 10.1016/j.jns.2012.03.006 22482825

[19] Steinberg A, Fourier C, Ran C, Waldenlind E, Sjöstrand C, Belin AC. Cluster headache—clinical pattern and a new severity scale in a Swedish cohort. Cephalalgia. 2018;38(7):1286-1295. doi: 10.1177/0333102417731773 28906127

[20] Duarte LL, Menna-Barreto L, Miguel MAL, et al. Chronotype ontogeny related to gender. Braz J Med Biol Res. 2014;47(4):316-320. doi: 10.1590/1414-431X20143001 24714814 PMC4075295

[21] Randler C, Engelke J. Gender differences in chronotype diminish with age: a meta-analysis based on morningness/chronotype questionnaires. Chronobiology Int. 2019;36(7):888-905. doi: 10.1080/07420528.2019.1585867 31070061

[22] Manzoni GC, Taga A, Russo M, Torelli P. Age of onset of episodic and chronic cluster headache – a review of a large case series from a single headache centre. J Headache Pain. 2016;17(1):44. doi: 10.1186/s10194-016-0626-9 27102121 PMC4840133

[23] Van Vliet JA, Favier I, Helmerhorst FM, Haan J, Ferrari MD. Cluster headache in women: relation with menstruation, use of oral contraceptives, pregnancy, and menopause. J Neurol Neurosurg Psychiatry. 2006;77(5):690-692. doi: 10.1136/jnnp.2005.081158 16407458 PMC2117457

[24] Lund N, Petersen A, Snoer A, Jensen RH, Barloese M. Cluster Headache is associated with unhealthy lifestyle and lifestyle-related comorbid diseases: results from the danish cluster headache survey. Cephalalgia 2018;39(2):254-263. doi: 10.1177/0333102418784751 29933701

[25] O’Connor E, Simpson BS, Houlden H, Vandrovcova J, Matharu M. Prevalence of familial cluster headache: a systematic review and meta-analysis. J Headache Pain. 2020;21(1):37. doi: 10.1186/s10194-020-01101-w 32334514 PMC7183702

[26] Waung MW, Taylor A, Qualmann KJ, Burish MJ. Family history of cluster headache: a systematic review. JAMA Neurol. 2020;77(7):887-896. doi: 10.1001/jamaneurol.2020.0682 32310255 PMC7644512

[27] Lund NLT, Snoer AH, Jensen RH. The influence of lifestyle and gender on cluster headache. Curr Opin Neurol. 2019;32(3):443-448. doi: 10.1097/WCO.0000000000000680 30844861

[28] Manzoni GC, Micieli G, Granella F, Martignoni E, Farina S, Nappi G. Cluster headache in women: clinical findings and relationship with reproductive life. Cephalalgia. 1988;8(1):37-44. doi: 10.1046/j.1468-2982.1988.0801037.x 3359483

[29] Svensson DA, Ekbom K, Larsson B, Waldenlind E. Lifetime prevalence and characteristics of recurrent primary headaches in a population-based sample of Swedish twins. Headache. 2002;42(8):754-765. doi: 10.1046/j.1526-4610.2002.02177.x 12390638

[30] Nilsson S, Edvinsson L, Malmberg B, Johansson B, Linde M. A relationship between migraine and biliary tract disorders: findings in two Swedish samples of elderly twins. Acta Neurol Scand. 2010;122(4):286-294. doi: 10.1111/j.1600-0404.2009.01310.x 20047569

[31] Rasmussen BK, Jensen R, Schroll M, Olesen J. Epidemiology of headache in a general population-A prevalence study. J Clin Epidemiol. 1991;44(11):1147-1157. doi: 10.1016/0895-4356(91)90147-2 1941010

[32] Schwartz BS, Stewart WF, Simon D, Lipton RB. Epidemiology of tension-type headache. JAMA. 1998;279(5):381-383. doi: 10.1001/JAMA.279.5.381 9459472

[33] Krøll LS, Hammarlund CS, Westergaard ML, et al. Level of physical activity, well-being, stress and self-rated health in persons with migraine and co-existing tension-type headache and neck pain. J Headache Pain. 2017;18(1):46. doi: 10.1186/s10194-017-0753-y 28421374 PMC5395520

[34] Ofte HK, Berg DH, Bekkelund SI, Alstadhaug KB. Insomnia and periodicity of headache in an arctic cluster headache population. Headache: J Head Face Pain. 2013;53(10):1602-1612. doi: 10.1111/head.12241 24266336

[35] Pringsheim T. Cluster headache: evidence for a disorder of circadian rhythm and hypothalamic function. Can J Neurol Sci. 2002;29(1):33-40. doi: 10.1017/S0317167100001694 11858532

[36] Gil-Gouveia R, Pavaño-Martins I, Neves-Costa A, Pedroso D, Barros A, Moita LF. Clock gene expression in cluster headache. Cephalagia. 2019;39(1_suppl):1-337. 10.1177/03331024241247845 38676534

[37] Su S, Li X, Xu Y, McCall Wv, Wang X. Epidemiology of accelerometer-based sleep parameters in US school-aged children and adults: NHANES 2011-2014. Sci Rep. 2022;12(11):7680-7688. doi: 10.1038/s41598-022-11848-8 35538108 PMC9090869

[38] Krishnan V, Collop NA. Gender differences in sleep disorders. Curr Opin Pulm Med. 2006;12(6):383-389. doi: 10.1097/01.mcp.0000245705.69440.6a 17053485

[39] Zhang B, Wing YK. Sex differences in insomnia: a meta-analysis. Sleep. 2006;29(1):85-93. doi: 10.1093/SLEEP/29.1.85 16453985

[40] Schürks M, Kurth T, de Jesus J, Jonjic M, Rosskopf D, Diener HCC. Cluster headache: clinical presentation, lifestyle features, and medical treatment. Headache. 2006;46(8):1246-1254. doi: 10.1111/j.1526-4610.2006.00534.x 16942468

[41] Rozen TD. Inhaled oxygen for cluster headache: efficacy, mechanism of action, utilization, and economics. Curr Pain Headache Rep. 2012;16(2):175-179. doi: 10.1007/s11916-012-0246-2 22286556

[42] Bangasser DA, Eck SR, Telenson AM, Salvatore M. Sex differences in stress regulation of arousal and cognition. Physiol Behav. 2018;187:42-50. doi: 10.1016/j.physbeh.2017.09.025 28974457 PMC6402500

[43] Schoonman GG, Evers DJ, Terwindt GM, Van Dijk JG, Ferrari MD. The prevalence of premonitory symptoms in migraine: a questionnaire study in 461 patients. Cephalalgia. 2006;26(10):1209-1213. doi: 10.1111/j.1468-2982.2006.01195.x 16961788

[44] Cho S, Cho SJ, Lee MJ, et al. Clinical characteristics of pre-attack symptoms in cluster headache: a large series of Korean patients. Cephalalgia. 2020;41(2):227-236. doi: 10.1177/0333102420966983 33086875

[45] Steinberg A, Josefsson P, Alexanderson K, Sjöstrand C. Cluster headache: prevalence, sickness absence, and disability pension in working ages in Sweden. Neurology. 2019;93(4):E404–E413. doi: 10.1212/WNL.0000000000007787 31213498

